# Interrogating the haemodynamic effects of haemodialysis arteriovenous fistula on cardiac structure and function

**DOI:** 10.1038/s41598-021-97625-5

**Published:** 2021-09-13

**Authors:** Sokratis Stoumpos, Alastair Rankin, Pauline Hall Barrientos, Kenneth Mangion, Ellon McGregor, Peter C. Thomson, Karen Stevenson, Paul Welsh, Ram Kasthuri, David B. Kingsmore, Giles Roditi, Patrick B. Mark

**Affiliations:** 1 Institute of Cardiovascular and Medical Sciences, University of Glasgow, Glasgow, UK; 2 Renal & Transplant Unit, Queen Elizabeth University Hospital, Glasgow, UK; 3 Department of Radiology, Queen Elizabeth University Hospital, Glasgow, UK; 4 Department of Cardiology, Queen Elizabeth University Hospital, Glasgow, UK

**Keywords:** Haemodialysis, Cardiac hypertrophy

## Abstract

Arteriovenous fistula (AVF) is the preferred type of vascular access for maintenance haemodialysis but it may contribute to maladaptive cardiovascular remodelling. We studied the effect of AVF creation on cardiac structure and function in patients with chronic kidney disease (CKD). In this prospective cohort study patients with CKD listed for first AVF creation underwent cardiac magnetic resonance (CMR) imaging at baseline and at 6 weeks. All participants had ultrasound measurements of fistula blood flow at 6 weeks. The primary outcome was the change in left ventricular (LV) mass. Secondary outcomes included changes in LV volumes, LV ejection fraction, cardiac output, LV global longitudinal strain and N-terminal-pro B-type natriuretic peptide (NT-proBNP). A total of 55 participants were enrolled, of whom 40 (mean age 59 years) had AVF creation and completed both scans. On the second CMR scan, a mean increase of 7.4 g (95% CI 1.1–13.7, p = 0.02) was observed in LV mass. Significant increases in LV end-diastolic volumes (p = 0.04) and cardiac output (p = 0.02) were also seen after AVF creation. No significant changes were observed in LV end-systolic volumes, LV ejection fraction, NT-proBNP and LV global longitudinal strain. In participants with fistula blood flows ≥ 600 mL/min (n = 22) the mean increase in LV mass was 15.5 g (95% CI 7.3–23.8) compared with a small decrease of 2.5 g (95% CI − 10.6 to 5.6) in participants with blood flows < 600 mL/min (n = 18). Creation of AVF for haemodialysis resulted in a significant increase of LV myocardial mass within weeks after surgery, which was proportional to the fistula flow.

## Introduction

Establishment of a functioning arteriovenous fistula (AVF) has been the mainstay of haemodialysis (HD) treatment due to its durability, delivery of adequate solute clearance and low risk of infection. However, creation of an AVF is not benign and may have deleterious effects on cardiac structure and function^1,2^. Creation of an AVF establishes a shunt from the arterial to the venous circulation, and exposes the low pressure, high capacitance venous system to the high pressure, low capacitance arterial system. Immediately following creation, there is an associated increase in cardiac output and a decrease in subendocardial perfusion as a consequence of reduced systemic vascular resistance, increased myocardial contractility, and an increase in stroke volume and heart rate^3,4^. Over the following weeks, circulating blood volume increases in conjunction with increases in atrial and brain natriuretic peptides^1,5^. As the fistula increases in size and blood flow, there are further increases in left ventricular (LV) filling pressure and subsequent changes on atrial and ventricular chamber dimensions and function^1,2,5,6^. The physiological consequences may even progress till there is myocardial decompensation, left ventricle dilatation, and a decline in ejection fraction, eventually leading to LV hypertrophy (LVH) or heart failure^1,7^. All these changes are additive to underlying pre-existing LVH, dilatation, and dysfunction caused by progressive uraemic cardiomyopathy and may be associated with adverse outcomes in dialysis patients^6^.

Maladaptive cardiac remodelling and, in particular, increased LV mass is an independent predictor of adverse cardiovascular events in CKD and has been used as a surrogate end point^8^. In the haemodialysis population, a study of adults with no history of congestive heart failure found that each 1 g/m^2^ increment in LV mass index was associated with a 62% increased risk of fatal and non-fatal cardiovascular events^9^. Despite its detrimental effects when present, the impact of LVH regression on mortality remains uncertain. An elegant multifactorial interventional study by London et al. has demonstrated that a 10% decrease in LV mass translated into a 28% decrease in cardiovascular mortality over a 5-year period^10^. Foley et al. found that improvements in LV mass over a 1-year period after the initiation of dialysis were associated with a reduced likelihood of cardiac failure but notably not with mortality risk^11^. Patients particularly susceptible to these effects are the ones with pre-existing heart disease and high flow fistulas. Fistulas with flows exceeding 2 L/min and upper arm fistulas are traditionally at increased risk for the development of heart failure^2,12–14^.

The effects of AVF creation on functional and structural cardiac parameters have been previously studied. Using cardiac magnetic resonance (CMR) imaging in 24 patients with CKD stage 5 undergoing fistula creation, Dundon et al*.*^15^ showed a mean increase of 13% in LV mass, 21% in LV end-systolic volumes, and 25% in cardiac output 6 months after AVF creation.

Typically, arteriovenous fistulas attain 40–60% of maximum blood flow within 1 day of creation, and reach their maximum flow as early as 6 weeks after creation^16^. Parallel to the vascular remodelling in the fistula arm, creation of a non-physiologic shunt initiates the cascade of haemodynamic changes in the heart muscle, thus, perhaps structural changes and LVH develop quickly despite the widely held belief that changes evolve gradually. Data show that the mean brachial artery flow increases from 56 mL/min before to 365 mL/min 1 day after and to 720 mL/min 28 days after AVF creation^17^ and cardiac output increases by 15% in 14 days^1^.

The aims of this study were two-fold: (i) to investigate myocardial changes early after AVF creation and (ii) to determine whether myocardial changes are proportional to the AVF blood flow. To address these questions, a prospective study using CMR imaging to evaluate changes on cardiac structure and function 6 weeks after AVF surgery in patients with advanced CKD was performed.

## Results

### Participant characteristics

**Figure 1:**
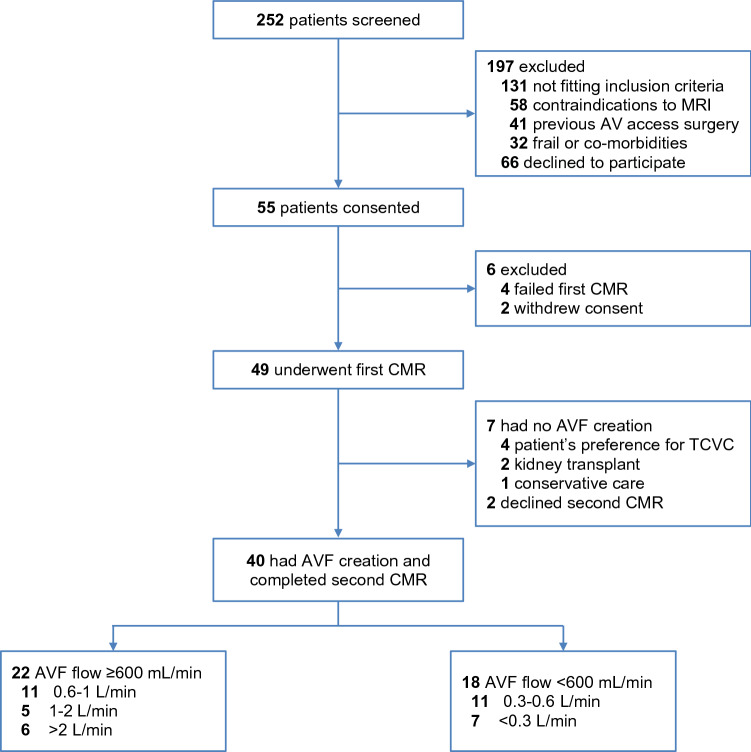
Flowchart showing recruitment and arteriovenous fistula (AVF) groups.

From December 2, 2016, to August 20, 2018, 252 participants were screened and 55 were enrolled (Fig. 1). Of the 197 subjects who were excluded, 131 were found to be ineligible for the study. The reasons for exclusion included contraindications to MRI (n = 58), previous AV access surgery (n = 41), and frailty (n = 32) (Supplementary Table S1). Of the 49 participants who underwent the first CMR scan, 7 had no AVF surgery, and 2 withdrew consent, declining the second scan. In total, 40 participants completed the study and were included in the analysis of primary and secondary outcomes. Twenty-two had brachial artery flow rates ≥ 600 (1386 ± 785) mL/min and 18 had flows < 600 (266 ± 197) mL/min at 6 weeks (Fig. 1).

The baseline characteristics of the study participants are shown in Table 1. There were no differences between the 2 groups in the location of AVF, baseline eGFR, and antihypertensive medication use. Antiplatelet use was more common in the higher flow AVF group, and 3 subjects (all in the higher flow group) were started on antiplatelet therapy post-AVF surgery. The median duration between CMR scans in the higher flow group was 63 days (interquartile range 51–115 days) and in the lower flow group was 51 days (interquartile range 46–63 days; p = 0.14). Three participants started dialysis within the 6-week period between the two scans (all via central venous catheters). The baseline cardiac parameters of the 2 groups were well matched (Supplementary Table S2).

**Table 1:** Characteristics of the study participants at baseline.

	All (n = 40)	AVF flow ≥ 600 mL/min (n = 22)	AVF flow < 600 mL/min (n = 18)	p value
Age, years	59 ± 13^a^	59 ± 15	60 ± 10	0.80
Female sex, n (%)	19 (48)	9 (41)	10 (56)	0.53
Time between first scan to AVF creation, days^b^	9 (4–23)	14 (5–74)	7 (2–17)	0.13
Time between first and second scan, days^b^	58 (47–85)	63 (51–115)	51 (46–63)	0.14
Time between AVF creation to second scan, days^b^	44 (41–49)	45 (41–49)	43 (41–46)	0.60
Diabetes mellitus, n (%)	15 (38)	6 (27)	9 (50)	0.19
Hypertension, n (%)	34 (85)	17 (77)	17 (94)	0.20
Ischaemic heart disease, n (%)	7 (18)	5 (23)	2 (11)	0.43
Peripheral vascular disease, n (%)	6 (15)	3 (14)	3 (17)	1.0
Smoking, n (%)^c^	22 (55)	12 (55)	10 (56)	1.0
**Location of AVF, n (%)**				0.11
Forearm AVF	16 (40)	6 (27)	10 (56)	
Upper arm AVF	24 (60)	16 (73)	8 (44)	
Serum creatinine, μmol/L^d^	458 ± 127	486 ± 140	394 ± 56	0.14
Estimated GFR, mL/min/1.73 m^2d^	12 ± 3	11 ± 2	13 ± 3	0.24
Dialysis, n (%)	17 (43)	7 (32)	10 (56)	0.20
**Medications, n (%)**				
ACEi or ARB	13 (33)	6 (27)	7 (39)	0.51
Beta blocker	24 (60)	12 (55)	12 (67)	0.53
Statin	25 (63)	16 (73)	9 (50)	0.19
Aspirin or Clopidogrel	19 (48)	15 (68)	4 (22)	0.005

*AVF* arteriovenous fistula, *GFR* glomerular filtration rate calculated using Chronic Kidney Disease Epidemiology Collaboration (CKD EPI) formula, *ACEi* angiotensin converting enzyme inhibitor, *ARB* angiotensin II receptor blocker.

^a^Plus-minus values are mean ± SD.

^b^Median (IQR).

^c^Current or previous.

^d^Includes 23 non-dialysis participants.

### Primary outcome

The primary end point showed a mean increase of 7.4 g (95% CI 1.1–13.7, p = 0.02) in LV mass. To adjust for body surface area, change in LV mass index was also calculated. An increase of 5.1 g/m^2^ (95% CI 1.6–8.6, p = 0.005) was found.

**Figure 2:**
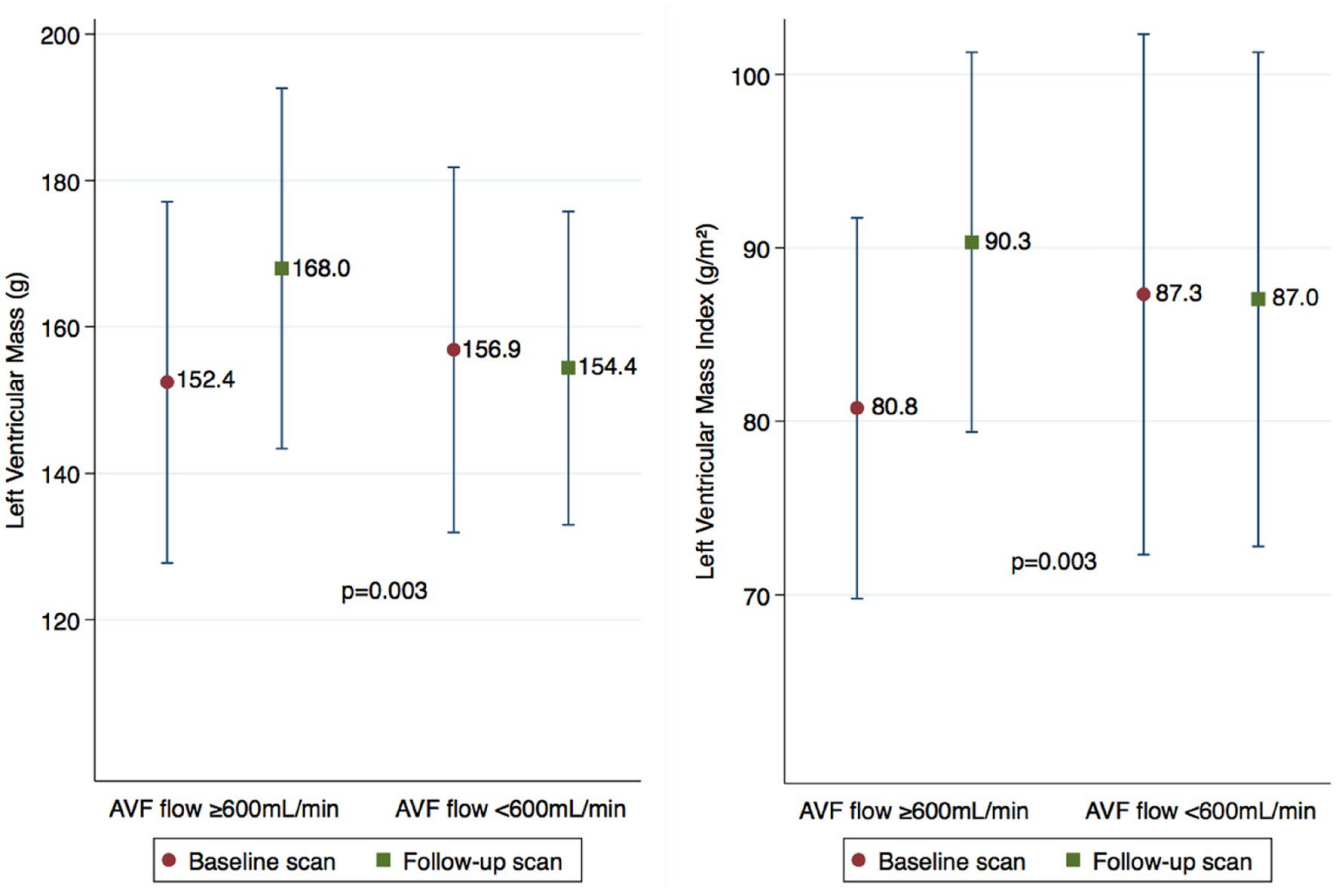
Difference in the means of left ventricular (LV) mass (left) and LV mass index (right) between the 2 scans according to the brachial artery blood flow (p = 0.003 for the difference between groups). This figure shows the means in 40 subjects who completed the second cardiac magnetic resonance scan, 22 with blood flows ≥ 600 mL/min and 18 with blood flows < 600 mL/min. Change in means and the 95% CI show a significant statistical difference between groups (p = 0.003) with an independent t-test.

In the higher flow group the mean increase in LV mass was 15.6 g (95% CI 7.3–23.8) compared with a small decrease of 2.5 g (95% CI − 10.6 to 5.6) in the lower flow group (p = 0.003 for comparison; Fig. 2). For the LV mass index the mean increase was 9.5 g/m^2^ (95% CI 5.2–14.0) in the higher flow group compared with a small decrease of 0.3 g/m^2^ (95% CI − 5.2 to 4.6) in the lower flow group (p = 0.003 for comparison; Fig. 2).

**Figure 3:**
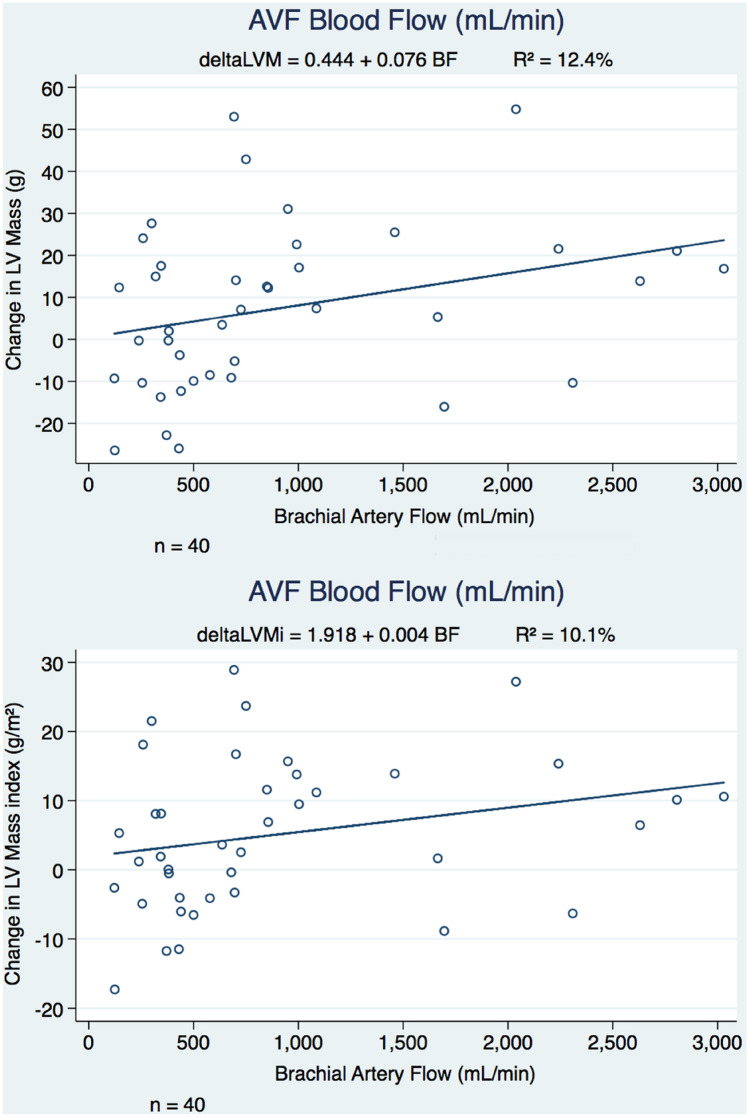
Effect of arteriovenous fistula (AVF) creation based on AVF blood flow. Linear regression analysis looking at change in LV mass (above) and LV mass index (below) in study participants with AVF creation. Increases in LV mass and LV mass index were more pronounced in those with higher fistula flows at 6 weeks after surgery.

Linear regression analyses undertaken showed that increase in the LV mass and LV mass index was more pronounced in subjects with higher flow fistulas (Fig. 3) and in subjects with lower LV mass at baseline (Supplementary Fig. S1). In the higher flow group, increase in LV mass was demonstrated in all except 2 subjects.

### Secondary outcomes

**Figure 4:**
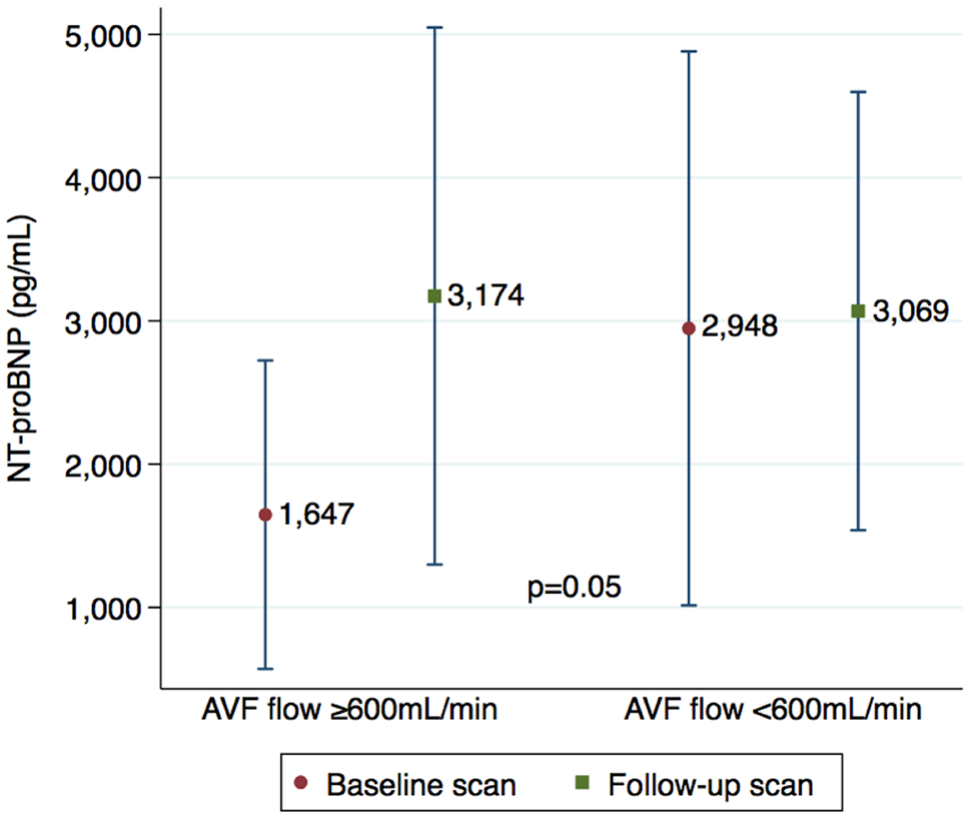
Effect of arteriovenous fistula (AVF) creation on N-terminal-pro B-type natriuretic peptide (NT-proBNP) levels. Changes in the blood NT-proBNP levels (picograms per millilitre) performed at the time of cardiac magnetic resonance scans according to the brachial artery blood flows (≥ 600 mL/min vs < 600 mL/min), showing a significant increase in the higher flow group compared with the lower flow AVF group (p = 0.05).

Table 2 shows the changes observed for other ventricular indexes and atrial size, including LV end-diastolic volume, LV end-systolic volume, LV global longitudinal strain, cardiac output, cardiac index, LA volume, and septal thickness. The mean NT-proBNP levels for all subjects were elevated at baseline (Table 2) and increased further in the higher flow AVF group (p = 0.05 for comparison; Fig. 4). There was a drop in diastolic blood pressure between CMR scans (p = 0.03) but no changes in systolic blood pressure, body weight and haematocrit were observed (Table 2).

**Table 2:** Summary of changes in cardiac magnetic resonance-derived cardiac indexes, clinical and laboratory parameters.

	All (n = 40)			AVF flow ≥ 600 mL/min (n = 22)			AVF flow < 600 mL/min (n = 18)		
	Baseline	Follow-up	p value	Baseline	Follow-up	p value	Baseline	Follow-up	p value
LV mass, g	154.4 ± 52.6^a^	161.8 ± 50.2	0.02	152.4 ± 55.6^a^	168.0 ± 55.6	< 0.001	156.9 ± 50.2	154.4 ± 43.0	0.52
LV mass index, g/m^2^	83.7 ± 27.1	88.8 ± 26.2	0.005	80.8 ± 24.7	90.3 ± 24.7	< 0.001	87.3 ± 30.2	87.0 ± 28.6	0.91
LV end-diastolic volume, mL	155.8 ± 55.1	165.3 ± 49.6	0.04	167.5 ± 58.7	182.0 ± 54.5	0.03	141.5 ± 48.2	145.0 ± 34.2	0.61
LV end-systolic volume, mL	51.3 ± 27.6	55.4 ± 27.1	0.12	57.2 ± 31.7	63.5 ± 32.8	0.10	44.1 ± 20.0	45.5 ± 13.0	0.70
LV ejection fraction, %	67.9 ± 7.8	67.1 ± 7.2	0.52	66.7 ± 8.1	66.1 ± 7.4	0.66	69.2 ± 7.6	68.4 ± 7.0	0.65
LV cardiac output, L/min	6.9 ± 2.1	7.5 ± 2.0	0.02	7.2 ± 1.9	8.2 ± 1.9	0.007	6.5 ± 2.2	6.8 ± 1.8	0.61
LV cardiac index, L/min/m^2^	3.8 ± 1.1	4.2 ± 1.1	0.009	3.9 ± 1.0	4.5 ± 1.2	0.002	3.6 ± 1.1	3.8 ± 0.9	0.53
LV global longitudinal strain, %	− 15.3 ± 2.3	− 15.7 ± 2.5	0.21	− 15.9 ± 1.9	− 16.0 ± 2.0	0.93	− 14.5 ± 2.6	− 15.2 ± 3.1	0.04
LA volume, mL	85.9 ± 40.1	93.4 ± 34.7	0.10	86.6 ± 43.3	99.3 ± 37.8	0.05	84.9 ± 37.0	86.2 ± 30.1	0.85
LA volume index, mL/m^2^	46.9 ± 21.0	52.1 ± 20.0	0.04	46.6 ± 21.9	54.5 ± 19.9	0.03	47.3 ± 20.6	49.2 ± 20.4	0.59
Septal thickness, mm	9.0 ± 2.9	8.5 ± 2.9	0.33	8.5 ± 2.9	7.8 ± 3.0	0.23	9.5 ± 2.9	9.5 ± 2.6	0.90
Body weight, kg	74.7 ± 16.7	73.3 ± 15.2	0.74	75.3 ± 17.2	73.6 ± 15.2	0.76	73.9 ± 16.7	73 ± 15.7	0.89
Systolic BP, mmHg	154.3 ± 29.8	147 ± 30.0	0.35	149.8 ± 24.0	146.2 ± 21.1	0.64	160.1 ± 36.3	148.2 ± 39.7	0.43
Diastolic BP, mmHg	76.3 ± 13.9	69.4 ± 10.5	0.03	77.3 ± 14.9	70.2 ± 12.2	0.14	74.9 ± 12.9	68.4 ± 8.0	0.13
Hematocrit, %	32.7 ± 5.2	32.4 ± 4.3	0.85	32.0 ± 4.5	31.5 ± 4.0	0.70	33.4 ± 5.9	33.6 ± 4.5	0.93
Serum creatinine, μmol/L^b^	458 ± 127	487 ± 161	0.53	486 ± 140	521 ± 180	0.57	394 ± 56	408 ± 53	0.65
Estimated GFR, mL/min/1.73 m^2b^	11.5 ± 2.6	10.3 ± 4.0	0.25	11.1 ± 2.3	9.5 ± 4.3	0.26	12.6 ± 3.0	11.9 ± 2.6	0.71
NT-proBNP, pg/mL	2390 ± 2513	3114 ± 2360	0.08	1647 ± 1401	3174 ± 2439	0.02	2948 ± 3043	3069 ± 2408	0.82
hs-cTnI, pg/mL	13.1 ± 11.2	12.0 ± 9.1	0.36	12.4 ± 12.1	12.7 ± 8.9	0.89	13.7 ± 10.9	11.4 ± 9.6	0.21

*AVF* arteriovenous fistula, *LA* left atrial, *LV* left ventricular, *BP* blood pressure, *GFR* glomerular filtration rate calculated using Chronic Kidney Disease Epidemiology Collaboration (CKD EPI) formula, *NT-proBNP* N-Terminal-pro B-type Natriuretic Peptide, *hs-cTnI* high-sensitivity cardiac Troponin I.

^a^Plus-minus values are mean ± SD.

^b^Includes 23 non-dialysis participants.

## Discussion

In this prospective observational study we have examined the effect of AVF creation on LV mass in patients with advanced CKD using a high fidelity cardiac imaging modality (CMR). There was a substantial increase in LV mass observed on the second CMR scan after an average time of 6.3 weeks from AVF surgery. The effect was proportional to the brachial artery flow rate, with a 10.2% increase in LV mass in participants with flows exceeding 600 mL/min compared with a small decrease in those with lower blood flows. To the best of our knowledge this is the first study demonstrating such early changes in LV mass, and linking the early AVF flow to the change in LV mass.

Arteriovenous fistula surgery is associated with adaptive cardiac remodelling necessary to accommodate the increase in cardiac output sufficient to service the fistula, whilst maintaining systemic blood supply. In a prospective study of 43 pre-dialysis patients, AVF formation resulted in a sustained reduction in arterial stiffness and blood pressure as well as an increase in cardiac output and LV ejection fraction using echocardiography^18^. In another prospective echocardiographic study of 47 patients, LV mass increased by 12.2 ± 32% one year post-AV access creation^19^. With AVF creation, we observed a 4.8% increase in LV mass, 6.1% increase in LV end-diastolic volume, 8.7% increase in LA volume and 8.7% increase in cardiac output. In patients with fistula flows ≥ 600 mL/min, the increases were 10.2%, 8.7%, 14.7% and 13.9%, respectively, and there was also an increase in NT-proBNP levels. Linear regression analysis showed that increase in the LV mass and LV mass index was more pronounced in subjects with lower LV mass at baseline, and in the higher flow group, increase in LV mass was demonstrated in all except 2 subjects. There are no previous studies examining the cardiac changes shortly after AVF creation for comparisons of our findings.

The typical blood flow in the brachial artery is approximately 50 mL/min, which rapidly increases 10- to 20-folds after the fistula is created^17^. Upper arm fistulas tend to have the highest blood flow rates and in fact, access flow has been shown to be up to twice as high in upper compared with forearm fistulas^2,16^. Consequently, the incidence of heart failure has been shown to be much higher in patients with a brachiocephalic compared to those with a radiocephalic AVF (40 vs 8%)^12^. In this study, 67% of the upper arm fistulas had flow rates exceeding 600 mL/min compared with 38% of the forearm fistulas and regression analysis showed that increases in the LV mass expectedly were proportional to the brachial artery flow.

Brain natriuretic peptide and its inactive N-terminal fragment have been studied extensively in patients with advanced CKD, and both markers have been shown to be strong predictors of cardiovascular morbidity and mortality^20,21^. They are thought to be secreted in greater quantities in response to increases in myocardial wall stretch^22^. Population-based studies suggest that plasma levels of brain natriuretic peptide and NT-proBNP are useful screening tests for heart failure and asymptomatic LV dysfunction^1,23^. In a short-term prospective study of 16 CKD patients both atrial natriuretic peptide (ANP) and BNP levels were maximally increased (by 48% and 68%, respectively) 10 days after AVF creation^1^. In another prospective study of 47 patients, plasma NT-proBNP levels increased by 170 ± 465% one year post-AV access creation^19^. The cardiac structural changes coupled with a rise in validated biomarkers of cardiac remodelling, such as NT-proBNP, suggest that observed changes could translate into clinical outcomes. To this end, the significant increases in LV and LA volumes in keeping with a rise in NT-proBNP in the higher flow AVF group may add important prognostic information. In addition, the minimal changes in LV mass, chamber volumes and NT-proBNP in the lower flow group are physiologically plausible and further strengthen our findings.

A recent meta-analysis of 14 studies showed that creation of AVF leads to modest but significant decreases in blood pressure in patients with advanced CKD^24^, with a preferential reduction in diastolic blood pressure^25^. It is assumed that the reduction in diastolic blood pressure may have been related to changes in arterial compliance and drop in systemic vascular resistance resulting from the complex effects of the fistula-related haemodynamic changes. A decrease in diastolic but no significant change in systolic blood pressure was seen in this study cohort. However, this study was powered to observe the change in CMR-derived LV mass and was not specifically powered to study the changes in blood pressure.

We have demonstrated that AVF creation may increase CMR-derived LV mass in patients with advanced CKD, particularly if ‘high flow’. Because of the short interval between the scans (median 8.3 weeks), the increase in LV mass observed is unlikely to be resulting from worsening of uraemia, plasma volume overload or changes in haematocrit and in fact serum creatinine, body weight and haematocrit did not change significantly between the scans. These results demonstrate that AVF creation, which is the cornerstone of haemodialysis treatment, has the potential to confer substantial cardiovascular risks in CKD patients, especially the ones with underlying cardiovascular disease. However, alternative vascular access choices have their own limitations. First, grafts are likely to induce similar haemodynamic changes and so may not reduce cardiovascular maladaptation. Second, catheters may have other risks, such as infection, that may result in adverse outcomes. Many unknowns remain, and several of those key questions were beyond the scope of this study. For example, baseline NT-proBNP levels, LV mass and LV mass index were higher in participants with blood flows < 600 mL/min, which is indicative of reduced LV compliance or “diastolic dysfunction”. A plausible alternative explanation of our findings is that cardiac stiffening and poor cardiac remodelling in this group resulted in lower AVF blood flows. Appropriately designed experimental studies are needed to infer causality. Also the long-term implications of the demonstrated changes in LV end-diastolic and LA volumes and BNP are unclear. Further work needs to be done to investigate whether they translate into adverse LV remodelling^26^ or systolic dysfunction and if so over what time period. Or whether they normalise with dialysis.

The strengths of this study include a prospective consecutive design, use of CMR as cardiac imaging modality, and standardised scanning protocols to limit variability. The study limitations included use of the modified Simpson’s method rather than the short axis stack of cines for estimation of LV parameters. We lack power and follow-up duration to detect clinically significant outcomes, such as cardiovascular events and patient survival. Although the cohort was a single-center experience with relatively small number of participants, our study group is representative of the typical CKD population referred for AVF creation. The right heart was not assessed, but the study was adequately powered to examine LV mass, which was our primary outcome.

We have shown that AVF creation is associated with a significant increase in LV myocardial mass, cardiac chamber dimensions and NT-proBNP levels. The changes are more pronounced in higher blood flow arteriovenous fistulas. Although these surrogate markers are strongly associated with cardiovascular outcomes, the impact of AVF creation on clinical and mortality outcomes remains to be proved.

## Methods

### Study protocol

A prospective observational single-centre study was conducted between December 2, 2016, and August 20, 2018. The study protocol (http://dx.doi.org/10.36399/gla.pubs.215112) was approved by the institutional review board (North of Scotland Research Ethics Committee reference number, 16/NS/0099) and registered with ClinicalTrials.gov (NCT02997046). Written informed consent was obtained from all participants, and the studies were performed according to hospital guidelines for diagnostic procedures.

### Study population

Pre-dialysis and dialysis patients (≥ 18 years of age) listed for creation of an autogenous forearm or upper arm AVF were eligible for enrollment. Dialysis patients were only included if they were dialysing via a tunnelled catheter and had no prior permanent access. Consecutive patients with CKD who attended the outpatient renal clinics were screened for eligibility. Exclusion criteria were (a) standard contraindications to MRI, (b) frail, elderly or participants with multiple or serious comorbidities, and (c) previous arteriovenous access creation (autogenous or synthetic graft) in either or both arms. All participants had CMR imaging followed by MR angiography with relevance to research questions out with the present study^27^.

### Study procedures

Baseline CMR imaging was performed (3.0 T Prisma, Magnetom; Siemens Healthineers, Erlangen, Germany) before undergoing AVF creation. Repeat CMR and duplex ultrasound (US) of the fistula arm was conducted 6 weeks after the creation of AVF.

All scans were performed following a standardised protocol (online Data Supplement, Supplementary Fig. S2). Clinical history and blood tests were performed at the time of the first and second CMR scans. Analyses of CMR-derived LV mass and chamber volumes was performed offline by a research fellow (A.R.) trained in CMR reporting who was blinded to all clinical information and analysed anonymised images in a random order (Supplementary Table S3). The ipsilateral brachial artery flow rate was measured by an interventional radiologist with more than 20 years experience in fistula imaging (R.K.) using a Philips iU22 colour duplex scanner (Philips Healthcare, Bothell, WA, USA). Participants were categorised into 2 groups based on brachial artery flow rate (≥ 600 mL/min vs < 600 mL/min). The threshold of 600 mL/min was used as a surrogate of maturation based on the “rule of 6 s” in fistula assessment at 6 weeks^28^. Brachial artery flows were assessed independently from the other cardiac parameters to ensure blinding. Serum N-terminal-pro B-type natriuretic peptide (NT-proBNP) and high-sensitivity cardiac Troponin I (hs-cTnI) were measured by immunoassays (Alere NT-proBNP and Stat high sensitive Troponin I, Architect, Abbott). Estimated glomerular filtration rate (eGFR) was calculated using the Chronic Kidney Disease Epidemiology Collaboration (CKD-EPI) equation^29^. Information on the history of diabetes mellitus, hypertension, ischaemic heart disease, and peripheral vascular disease was obtained from the electronic patient records.

### Outcomes

The primary outcome was change in CMR-derived LV mass in the total population. Secondary outcome measures included changes in LV end-diastolic and systolic volumes, left atrial (LA) volume, LV ejection fraction, cardiac output, cardiac index, LV global longitudinal strain, septal thickness, NT-proBNP, and hs-cTnI. Primary and secondary outcomes were also assessed for the blood flow subgroups.

### Statistical analysis

The sample size was calculated using data from a single-arm pilot study involving CMR imaging prior to and 6 months following fistula creation that demonstrated increases in LV mass with AVF creation^15^. Based on the increase in LV mass as the primary end point, power calculations (including accounting for a dropout rate of 15%) indicated that 49 subjects would provide a 7% difference in the LV mass from baseline to 6-week CMR with a power of > 80% and a 2-sided α level of 0.05 (online Data Supplement).

Descriptive statistics are expressed as means and standard deviations or medians and interquartile ranges for continuous measures as appropriate, and percentages are reported for categorical variables. We used independent sample t-tests and Pearson’s chi-squared tests to explore differences in baseline characteristics.

Independent t-tests were also used to compare the change in LV mass between the first and second scans for the total population and between the 2 groups. Two-sided tests were performed for all analyses, and the level of significance was set at p < 0.05. To estimate pre- versus post-AVF creation differences, the change between initial and subsequent measurements was calculated for each person. The mean change was compared between the 2 groups with an unpaired t-test. For comparisons of non-normally distributed data, Wilcoxon rank-sum and Wilcoxon signed rank tests were used. All statistical analyses were conducted with Stata/SE Statistical Software, version 15.0 (StataCorp, College Station, Tex).

## Supplementary Information

Supplementary Information.
