# Supplementary material for: Interrogating the haemodynamic effects of haemodialysis arteriovenous fistula on cardiac structure and function
Source: Sci Rep. 2021 Sep 13;11:18102. doi: 10.1038/s41598-021-97625-5 (PMC8437985; doi:10.1038/s41598-021-97625-5)
Supplement: Supplementary file 1 — Supplementary Information. [file 41598_2021_97625_MOESM1_ESM.docx]

Supplementary Material

List of investigators 3

Power Calculation 4

Supplementary Table 1 5

Supplementary Table 2 6

Supplementary Figure 1 7

Cardiac magnetic resonance imaging protocol 9

CMR image acquisition 9

CMR image analysis 9

Supplementary Figure 2 11

Supplementary Table 3 13

References 14

# List of investigators

Sokratis Stoumpos^1,2^, Alastair Rankin^1,2^, Pauline Hall Barrientos^3^, Kenneth Mangion^1,4^, Ellon McGregor^2^, Peter C Thomson^2^, Karen Stevenson^2^, Paul Welsh^1^, Ram Kasthuri^3^, David B Kingsmore^2^, Giles Roditi^3^, Patrick B Mark^1,2^

^1^BHF Cardiovascular Research Centre, University of Glasgow, Glasgow, UK

^2^Renal & Transplant Unit, Queen Elizabeth University Hospital, Glasgow, UK

^3^Department of Radiology, Queen Elizabeth University Hospital, Glasgow, UK

^4^Department of Cardiology, Queen Elizabeth University Hospital, Glasgow, UK

# Power Calculation

The sample size was calculated using the increase in LV mass as the primary end point and data from a single-arm pilot study involving CMR imaging prior to and 6 months following fistula creation. In that study, AVF creation was associated with a significant increase in LV mass of 13% (from 132±32 to 149±35g) over a period of 6 months(1). We made the assumption that the change in LV mass is more pronounced over the first weeks following AVF creation and we hypothesised that approximately half of the LV mass increment (6.5%) observed in 6 months, occurs within the first 6 weeks. Based on this assumption, the study would require a random sample of 42 subjects to achieve statistical power of 80% and a level of significance of 5% (two-sided), for detecting a mean of the differences of 8g between scans and a standard deviation of 18(2). To include a dropout rate of 15%, the recruitment target was increased to 49 subjects.

# Supplementary Table 1

**Table S1.** Reasons for exclusion from the study (N=131)

| **Contraindications to MRI (N=58)** |
| --- |
| 22 claustrophobia |
| 16 not fitting into the scanner due to obesity |
| 11 metallic objects in the body |
| 9 unable to lie flat (i.e. back pain, dyspnea) |
| **Previous AV access surgery (N=41)** |
| 24 left arm |
| 11 right arm |
| 6 both arms |
| **Frail or co-morbidities (N=32)** |
| 25 frail* |
| 4 active cancer |
| 2 dementia |
| 1 blind |
| *Scoring ≥5 in the Clinical Frailty Scale(3) |

# Supplementary Table 2

**Table S2.** Comparison of baseline cardiac parameters between patients with arteriovenous fistula (AVF) flow ≥600 mL/min versus <600 mL/min

| Cardiac indices | AVF flow ≥600 mL/min (n=22) | AVF flow <600 mL/min (n=18) | p value |
| --- | --- | --- | --- |
| LV mass, g | 152.4 ± 55.6* | 156.9 ± 50.2 | 0.79 |
| LV mass index, g/m^2^ | 80.8 ± 24.7 | 87.3 ± 30.2 | 0.45 |
| LV end-diastolic volume, mL | 167.5 ± 58.7 | 141.5 ± 48.2 | 0.14 |
| LV end-systolic volume, mL | 57.2 ± 31.7 | 44.1 ± 20.0 | 0.14 |
| LV ejection fraction, % | 66.7 ± 8.1 | 69.2 ± 7.6 | 0.32 |
| LV cardiac output, L/min | 7.2 ± 1.9 | 6.5 ± 2.2 | 0.32 |
| LV cardiac index, L/min/m^2^ | 3.9 ± 1.0 | 3.6 ± 1.1 | 0.45 |
| LV global longitudinal strain, % | -16.0 ± 1.9 | -14.5 ± 2.6 | 0.04 |
| LA volume, mL | 86.6 ± 43.3 | 84.9 ± 37.0 | 0.90 |
| LA volume index, mL/m^2^ | 46.6 ± 21.9 | 47.3 ± 20.6 | 0.91 |
| Septal thickness, mm | 8.5 ± 2.9 | 9.5 ± 2.9 | 0.28 |
| AVF, arteriovenous fistula; LA, left atrial; LV, left ventricular  *Plus-minus values are mean±SD | | | |

#
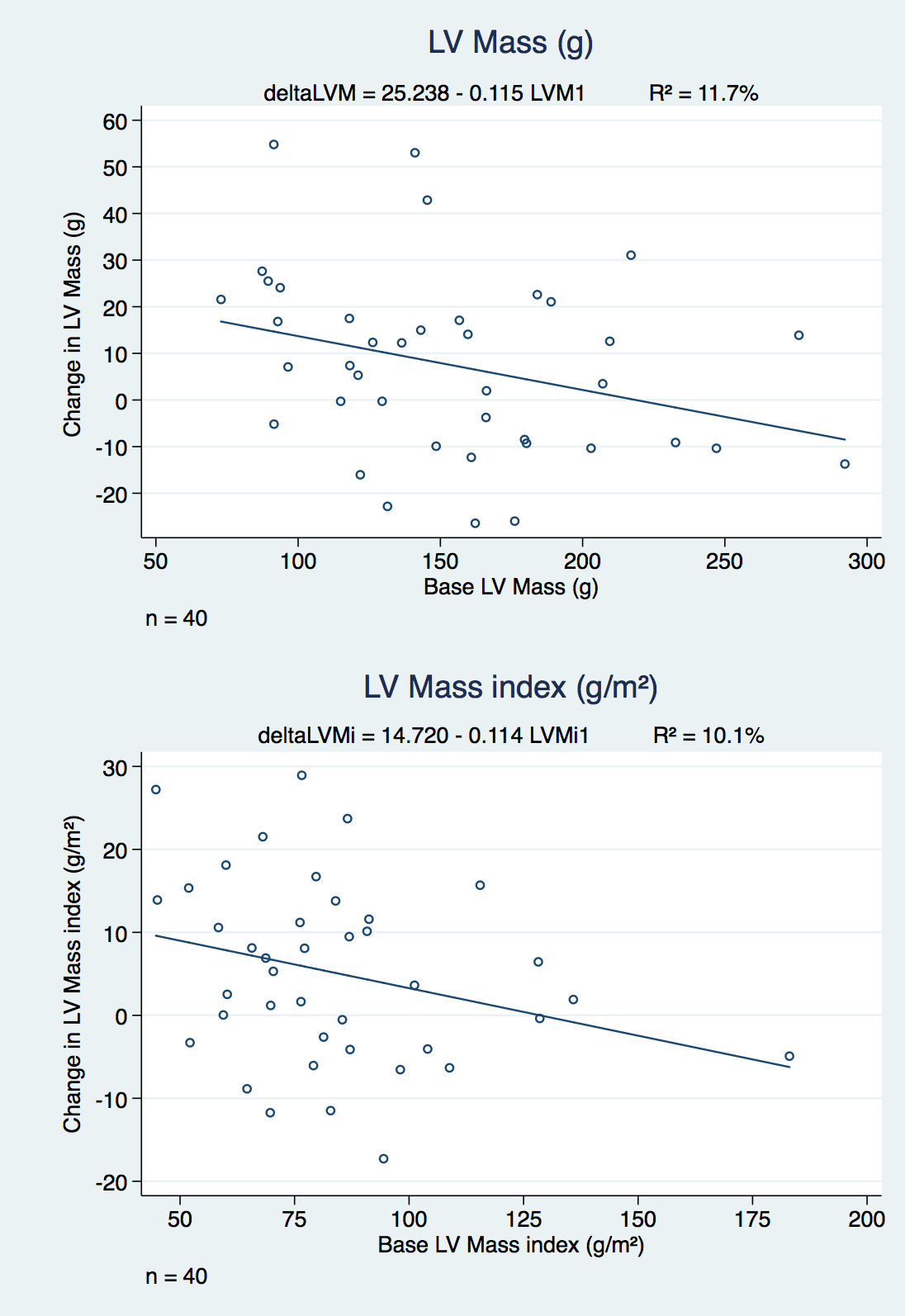


# Supplementary Figure 1

**Figure S1.** Effect of arteriovenous fistula (AVF) creation based on baseline left ventricular (LV) mass and LV mass index.

Linear regression analysis looking at change in LV mass (above) and LV mass index (below) in study participants with AVF creation. Increases in LV mass and LV mass index were more pronounced in those with lower LV mass and LV mass index in the baseline cardiac magnetic resonance imaging.

# Cardiac magnetic resonance imaging protocol

## CMR image acquisition

CMR acquisition was performed using a Siemens Prisma 3 Tesla scanner (Siemens Healthineers, Erlangen, Germany) with an 18-channel surface coil placed anteriorly and a 32-channel spine coil placed posteriorly. Scanning took place at the Clinical Research Imaging Facility based at the Queen Elizabeth University Hospital in Glasgow. A focused cardiac imaging protocol was used to allow additional sequences to be obtained with relevance to research questions outwith the present study(4). Following the acquisition of localiser images, balanced steady state free precession sequence was used to acquire left ventricular (LV) imaging in two long axis planes (horizontal and vertical long axis), followed by a single mid LV short axis view, each with 25 phases. Images were obtained at end-expiration using retrospective electrocardiogram gating. Typical acquisition parameters were: pixel bandwidth 977 Hz/pixel, echo time = 1.51 ms, repetition time = 3.5 ms, flip angle = 50^0^, matrix = 256 x 205, field of view = 340mm, slice thickness 7 mm.

## CMR image analysis

CMR data were analysed using CVi 42 software (version 5.10, Circle Cardiovascular Imaging, Calgary, Alberta, Canada). A single observer (A.R.) analysed anonymised images in a random order. Ventricular endocardial and epicardial contours were manually drawn at end-diastole (Figure S2). Left ventricular endocardial contours were drawn at end-systole, which was deemed to be the phase with the smallest blood pool cavity. Papillary muscles were excluded from myocardial mass and included in volumes. Modified Simpson’s method of discs was used to calculate ventricular volumes and myocardial mass, with derivation of stroke volume (SV), ejection fraction (EF) and cardiac output (CO) from these measurements(5). Parameters of myocardial mass and ventricular volumes were indexed to body surface area (using a weight acquired immediately pre-scan). Left ventricular thickness was recorded as the maximum septal thickness measured perpendicular to the cavity on a mid-chamber short axis view, at the approximate level of the mitral valve leaflet tips. Left atrial (LA) volume was calculated using the biplane area-length method by manual planimetry of the left atrium in horizontal and vertical long axis views in the end-systolic phase immediately prior to mitral valve opening. Pulmonary veins and LA appendage were excluded from LA volume. For the purposes of strain measurements, the previously described delineated ventricular contours were automatically propagated throughout the cardiac cycle using the software’s machine-learning algorithms. Automated contours were individually checked and corrected, where necessary. Global left ventricular strain (longitudinal and radial) and global right ventricular strain (longitudinal and radial) were derived using a feature-tracking algorithm within the software’s tissue tracking module, with reporting of peak global values (%). Twenty participants (mean age, 58 ± 12years; 9 women) were selected at random for determination of intra-observer reproducibility. Intraclass correlation coefficients (ICCs) were 0.84–0.99 for all parameters, indicating excellent agreement (Table S3).

#
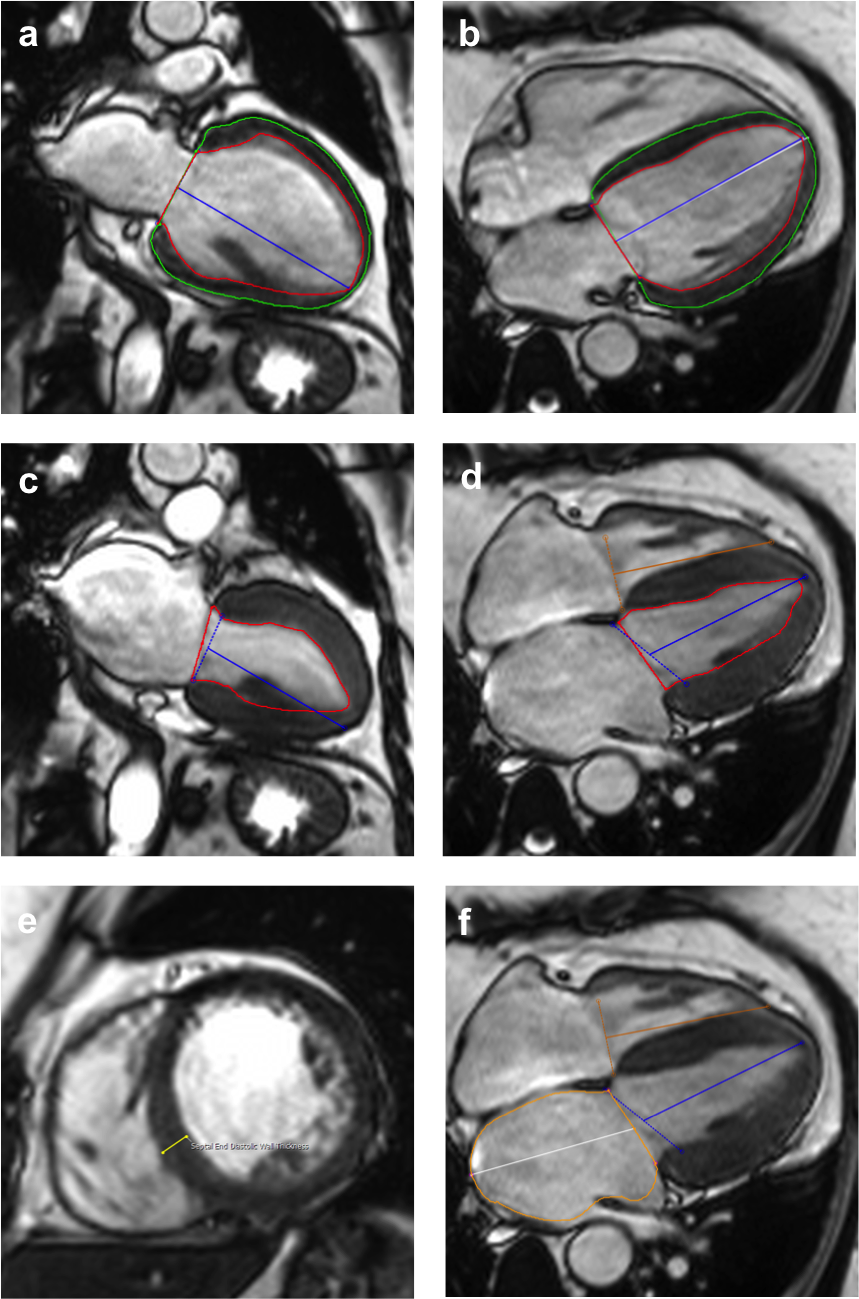


# Supplementary Figure 2

**Figure S2.** Cardiac magnetic resonance images representing the methodology of left ventricular (LV) assessment.

Biplanar LV mass was calculated by tracing endocardial and epicardial contours at end-diastole on vertical long axis (a) and horizontal long axis (b) views. Endocardial contours were then drawn on the phase with the smallest blood pool cavity representing end-systole (c, d), from which ejection fraction was derived. Left ventricular septal thickness was measured on a mid-chamber short axis view using a straight line drawn perpendicular to the endocardium at the thickest point of myocardium (e). Left atrial volume was measured by contouring the left atrium on the image immediately prior to mitral valve opening (f).

# Supplementary Table 3

**Table S3.** Intra-observer agreement for CMR parameters was assessed by ICC (two-way mixed effect, absolute agreement).

| CMR parameter | Intra-observer ICC |
| --- | --- |
| LV mass, g | 0.99 |
| LV end-diastolic volume, mL | 0.99 |
| LV end-systolic volume, mL | 0.90 |
| LV ejection fraction, % | 0.84 |
| LV global longitudinal strain, % | 0.97 |
| Septal thickness, mm | 0.89 |
| LA volume, mL | 0.99 |
| LA, left atrial; LV, left ventricular | |

# References

1. Dundon BK, Torpey K, Nelson AJ, Wong DT, Duncan RF, Meredith IT, et al. The deleterious effects of arteriovenous fistula-creation on the cardiovascular system: a longitudinal magnetic resonance imaging study. Int J Nephrol Renovasc Dis. 2014;7:337-45.

2. Dhand NK, Khatkar MS. Statulator: An online statistical calculator. Sample Size Calculator for Comparing Two Paired Means. 2014.

3. Rockwood K, Song X, MacKnight C, Bergman H, Hogan DB, McDowell I, et al. A global clinical measure of fitness and frailty in elderly people. CMAJ. 2005;173(5):489-95.

4. Stoumpos S, Tan A, Hall Barrientos P, Stevenson K, Thomson PC, Kasthuri R, et al. Ferumoxytol MR Angiography versus Duplex US for Vascular Mapping before Arteriovenous Fistula Surgery for Hemodialysis. Radiology. 2020;297(1):214-22.

5. Foley TA, Mankad SV, Anavekar NS, Bonnichsen CR, Morris MF, Miller TD, et al. Measuring Left Ventricular Ejection Fraction - Techniques And Potential Pitfalls. European Cardiology. 2012;8(2):108-14.
